# Patterns, Associated Factors and Plant Diversity Characteristics of *Solidago canadensis*-Invaded Communities in Jiangsu Province, China

**DOI:** 10.3390/plants15142198

**Published:** 2026-07-17

**Authors:** Huan Zhang, Zhen Wang, Yu Zhang, Zheng Zhang, Weiming Dai, Yujing Liu, Xiaoling Song, Sheng Qiang

**Affiliations:** College of Life Science, Nanjing Agricultural University, Nanjing 210095, China; sophia52024@163.com (H.Z.); 2022116012@stu.njau.edu.cn (Z.W.); zhangyu2013@njau.edu.cn (Y.Z.); zhangzheng@njau.edu.cn (Z.Z.); daiweimin4@njau.edu.cn (W.D.); liuyujing@njau.edu.cn (Y.L.); sxl@njau.edu.cn (X.S.)

**Keywords:** *Solidago canadensis*, Jiangsu Province, invasion intensity, plant diversity, habitat suitability, anthropogenic disturbance

## Abstract

*Solidago canadensis* L. is a regulated invasive alien species in China and was first recorded in the wild in Jiangsu Province. Despite widespread concern regarding its distribution and ecological associations, province-wide information on its current invasion status and associated plant community patterns in Jiangsu remains limited. To fill this gap, we conducted field surveys at 165 sites across Jiangsu Province and combined hierarchical clustering, plant-community diversity analysis, species-level analysis of accompanying-plant composition, redundancy analysis, and MaxEnt modeling to evaluate invasion-intensity patterns, associated environmental and anthropogenic factors, and plant-community responses in invaded communities. *Solidago canadensis* was recorded throughout the province, but invasion intensity was highest in southern Jiangsu. Hierarchical clustering classified the 165 sampling sites into three invasion-intensity groups. Group A (74 sites), concentrated in southern Jiangsu, was classified as the severe invasion group and showed generally lower plant diversity within the invaded-site dataset. Group B (60 sites) represented moderate invasion. Group C (31 sites), mainly distributed in northern and coastal Jiangsu, represented light invasion and retained relatively higher accompanying-plant diversity. Within *S. canadensis*-invaded communities, we documented 183 accompanying plant species from 43 families. Asteraceae and Poaceae were the most species-rich families, and annual or biennial herbs were the dominant life-form category. Species-level analysis further showed that accompanying-plant composition varied significantly along the invasion-intensity gradient (PERMANOVA, R^2^ = 0.035), suggesting that differences in *S. canadensis* dominance were associated with detectable shifts in local plant assemblages. Redundancy analysis indicated latitude, transportation route density, and floating population density were closely associated with variation in *S. canadensis* invasion indicators and associated community variables, with soil pH, precipitation of the driest month, GDP, and motor vehicle ownership also contributing to the observed pattern. MaxEnt modeling identified southern and central Jiangsu as the main climatically suitable areas. This pattern was broadly consistent with the concentration of higher invasion intensity in southern Jiangsu. Nevertheless, field records in northern Jiangsu suggest that areas with lower predicted climatic suitability should also remain under continued monitoring. These findings suggest the need for coordinated management, particularly along major transportation corridors and in highly disturbed urban habitats. Control efforts should combine zoning management, source control, repeated removal, and habitat restoration tailored to local conditions.

## 1. Introduction

Biological invasions are widely recognized as a major driver of biodiversity loss and ecosystem degradation worldwide [1]. Native to North America, *S. canadensis* has become a representative species in plant invasion research [2]. In China, its ecological impacts have been increasingly documented since the twentieth century, with early studies describing it as a rapidly spreading and highly noxious alien weed [3]. Consequently, it has been included in the “Second List of Invasive Alien Species in China” [4] and the “Key managed invasive alien species list” [5], with technical guidelines prioritizing its control in agricultural and forestry systems [6]. The species was first introduced as an ornamental plant in 1935 and was subsequently cultivated in gardens across southern Jiangsu and Shanghai. With urban expansion and land-use change, the species eventually escaped cultivation, establishing naturalized populations in peri-urban and other disturbed habitats [3].

Jiangsu Province is a highly urbanized and economically developed region with dense transportation networks and diverse ecological conditions [7]. These features, together with a long invasion history and intense anthropogenic disturbance, make Jiangsu a particularly important region for studying the invasion of *S. canadensis.* In the early 2000s, the provincial government invested substantial efforts to control this species [8]. In this region, the species predominantly colonizes areas with high human disturbance or neglected management, such as field margins, forest edges, wetland embankments, riverbanks, and abandoned urban lots [9]. In the rural–urban fringes of the Yangtze River Delta, populations have shifted from scattered individuals to locally monodominant stands, with potential consequences for local landscape integrity and ecosystem services [10]. While habitat suitability models predict that suitable areas for *S. canadensis* are concentrated mainly in central and eastern China [11], current evidence still relies heavily on localized case studies, and a province-wide understanding of its field occurrence, spatial variation in invasion intensity, and associated plant-community structure remains limited.

The spread of *S. canadensis* in Jiangsu is likely facilitated by both biological traits and human-mediated dispersal. Its early establishment in parks, campuses, and rural landscapes was promoted by horticultural cultivation, ornamental landscaping, and its use as a nectar source [12]. The species reproduces through both prolific seed production and clonal expansion via rhizomes. Its pappus-bearing achenes facilitate long-distance wind dispersal, while rhizomes promote local expansion and the formation of dense patches in disturbed habitats [13,14]. Linear corridors, including Yangtze River tributaries, canals, highways, railways, port shorelines, and shelterbelts, may further facilitate the dispersal of *S. canadensis* from southern Jiangsu into central and northern Jiangsu, particularly because transport corridors and vehicle movements can promote propagule dispersal of alien plants. In addition, trade and construction activities, especially the movement of contaminated soil, may also contribute to new occurrences at road intersections and construction sites [15]. Furthermore, nitrogen deposition has been reported to enhance its competitive ability and biomass accumulation, potentially facilitating its expansion [16,17]. Together, wind dispersal, vegetative expansion, and human-mediated transport provide plausible mechanisms for the spread of *S. canadensis* from initial occurrence sites to linear corridors and adjacent disturbed habitats across Jiangsu Province.

Beyond its distribution, *S. canadensis* invasion may also alter local plant communities. Recent studies have shown that *S. canadensis* invasion is often associated with changes in plant community structure [18] and soil microbial dynamics [19]. Moderate-to-severe *S. canadensis* invasions are often associated with simplified species composition and lower Shannon diversity, particularly in communities approaching monodominance [18]. These above-ground vegetation changes may interact with below-ground processes, as previous studies have shown that *S. canadensis*-invaded sites can exhibit altered nitrogen-fixing bacterial communities and changes in enzyme activity [20]. In addition, litter leachates and root exudates of *S. canadensis* can reshape soil bacterial, fungal, and actinomycete communities, potentially affecting nitrification, nutrient cycling, and soil multifunctionality [21,22]. Furthermore, legacy effects from established populations may facilitate re-invasion and enhance the competitive ability of *S. canadensis* [23]. Co-occurring alien Asteraceae species may further alter both plant and soil microbial diversity [24]. Together, these findings suggest that *S. canadensis* invasion may be associated with coordinated changes in above- and below-ground community attributes, although the strength and direction of these associations may vary across habitats and invasion stages.

Despite extensive research on its biological traits, invasion mechanisms, and ecological effects [25], most studies have focused on specific cities, habitat types, or ecological processes [26]. In Jiangsu, systematic field surveys based on consistent sampling designs across the entire province remain limited [24,27]. Therefore, this study investigated *S. canadensis*-invaded communities throughout Jiangsu Province to address three objectives: (1) to characterize province-wide patterns of invasion intensity; (2) to identify environmental and anthropogenic variables associated with its field distribution; and (3) to examine variation in accompanying-plant diversity and species composition along the invasion-intensity gradient. By integrating field-based invasion-intensity classification, community diversity and species-composition analyses, redundancy analysis, and climatic habitat suitability modeling, this study identifies key invasion areas and community patterns, thereby providing a basis for region-specific control measures and sustained long-term monitoring in the middle and lower Yangtze River region.

## 2. Results

### 2.1. Distribution Pattern of S. canadensis in Jiangsu Province

#### 2.1.1. Overall Occurrence of *S. canadensis*

**Figure 1:**
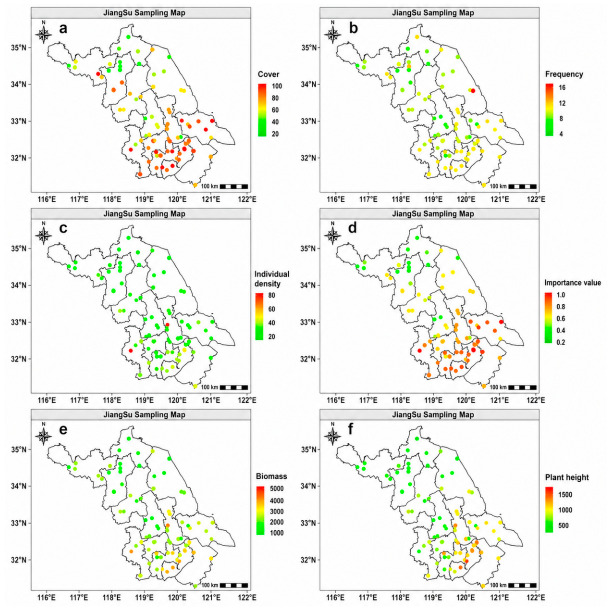
Spatial distribution of field-measured invasion indicators of *S. canadensis* in Jiangsu Province. (**a**) Cover (%); (**b**) Frequency; (**c**) Individual density; (**d**) Importance value; (**e**) Biomass (g m^−2^); (**f**) Plant height (cm).

The field survey of 165 sites across Jiangsu Province showed that *S. canadensis* occurred throughout the surveyed area, with generally higher population dominance in southern Jiangsu and lower invasion indicators in northern Jiangsu (Figure 1). Sites with higher cover, density, importance value, and biomass of *S. canadensis* were mainly associated with highly disturbed habitats, particularly those with dense road networks and strong anthropogenic disturbance. This pattern generally matches the known distribution of invasive plants in China [13,14]. In southern Jiangsu, the population-level invasion indicators of *S. canadensis* were relatively high. Compared with the provincial averages, the cover (17.90% higher), frequency (15.15% higher), individual density (46.66% higher), importance value (30.34% higher), and biomass (25.75% higher) of *S. canadensis* were elevated. In contrast, the total cover of accompanying plants was 48.32% lower than the provincial average, indicating reduced cover of co-occurring vegetation in areas where *S. canadensis* was more dominant.

In central Jiangsu, *S. canadensis* exhibited lower values than the provincial averages for cover (1.82% lower), frequency (7.08% lower), individual density (38.20% lower), and importance value (10.34% lower). However, plant height was slightly higher (2.51% above average), and the richness of accompanying species was 3.11% higher. In northern Jiangsu, the importance value (39.66% lower), cover (25.05% lower), frequency (16.46% lower), individual density (36.82% lower), and biomass (54.36% lower) were all markedly lower than the provincial averages. Meanwhile, the total cover of accompanying plants was 76.91% higher than the provincial average, representing the highest value among the three broad regions. These results indicate that northern Jiangsu had the lowest population dominance of *S. canadensis* among the surveyed regions.

#### 2.1.2. Regional Characteristics of *S. canadensis* Invasion

**Figure 2:**
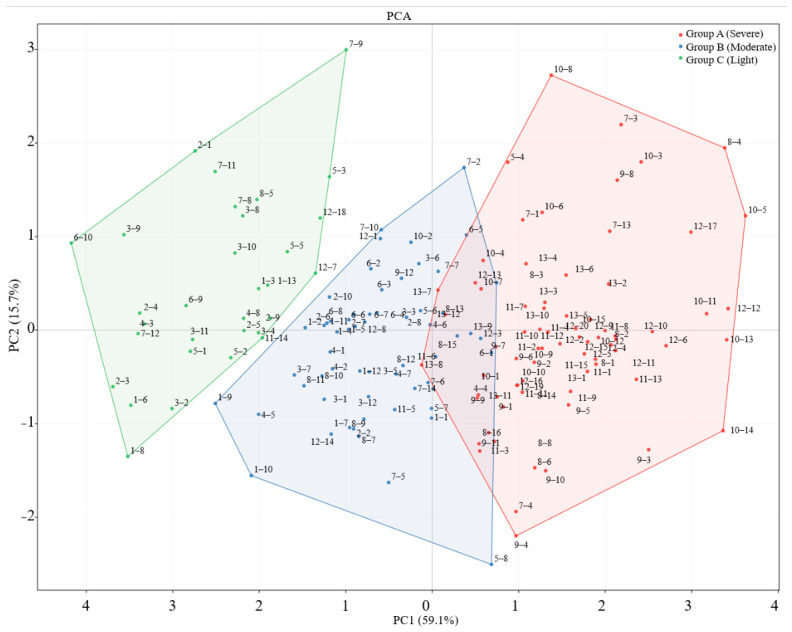
PCA ordination of sampling sites based on field-measured *S. canadensis* invasion indicators.

To further characterize regional variation in invasion intensity across Jiangsu Province, hierarchical clustering was applied to five standardized field-measured variables of *S. canadensis* at the plot scale, including plant height, cover, frequency, individual density, and biomass. Based on these variables, the 165 sampling sites were classified into three cluster-defined invasion-intensity groups. PCA was then used only to visualize the separation among the resulting clusters. The ordination plot showed that the three groups occupied relatively distinct regions in the ordination space (Figure 2).

The three invasion categories were defined by hierarchical clustering and then interpreted according to their overall population-level invasion indicators of *S. canadensis* (Table 1).

Group A (*n* = 74) consisted of sites with severe invasion. The plant height (109.35 ± 2.82 cm), cover (82.90 ± 1.36%), frequency (8.69 ± 0.12), and individual density (27.35 ± 1.60 plants m^−2^) were the highest among the three groups. The importance value, reported as an additional descriptor of population dominance, was also highest in this group (0.78 ± 0.02), indicating strong population dominance of *S. canadensis*. Biomass reached 688.08 ± 27.05 g m^−2^, further indicating the presence of dense and highly dominant *S. canadensis* populations in these areas. These sites were concentrated mainly in southern Jiangsu, particularly in Nanjing, Wuxi, Changzhou, Zhenjiang, and Suzhou, with additional occurrences in Nantong and Taizhou. The soils in these areas were mainly yellow-brown and alluvial sandy soils.

Group B (*n* = 60) consisted of sites with moderate invasion. Plant height (90.62 ± 2.55 cm), cover (64.73 ± 1.63%), frequency (7.18 ± 0.23), individual density (11.34 ± 0.62 plants m^−2^), biomass (350.26 ± 15.36 g m^−2^), and importance value were intermediate between those of Groups A and C. The accompanying vegetation remained present, although its cover was lower than in lightly invaded sites. Frequently recorded accompanying plant species included *Erigeron canadensis* L., *Digitaria sanguinalis* (L.) Scop., and *Causonis japonica* (Thunb.) Raf. Several other accompanying species, such as *S. viridis*, *H. scandens*, *S. faberi*, *G. soja*, *P. australis*, and *A. lavandulifolia*, were also recorded. This group was widely distributed across the province, with relatively high representation in Xuzhou, Yangzhou, Nantong, Suqian, Huai’an, Taizhou, and Lianyungang, and scattered occurrences in Yancheng, Wuxi, Changzhou, Zhenjiang, Nanjing, and Suzhou. The soil was mainly yellow-brown soil.

Group C (*n* = 31) consisted of lightly invaded sites. In this group, *S. canadensis* showed the lowest values for cover (32.66 ± 1.96%), frequency (4.40 ± 0.21), individual density (6.08 ± 0.51 plants m^−2^), and biomass (193.51 ± 20.81 g m^−2^), while plant height (86.06 ± 4.88 cm) was lower than in Group A. Importance value was also lowest in this group (0.31 ± 0.03). *C. dactylon* was the dominant accompanying plant species, while *S. viridis*, *H. scandens*, *S. faberi*, *C. rostellatum*, and *A. lavandulifolia* occurred as secondary species. Wetland or wetland-associated plants, including *P. australis*, *Miscanthus sacchariflorus* (Maxim.) Hack., and *Murdannia nudiflora* (L.) Brenan, were frequently observed. These sites were distributed mainly in northern and coastal Jiangsu, especially in Suqian, Lianyungang, Xuzhou, and Yancheng, with only scattered occurrences in Huai’an, Yangzhou, Taizhou, Nantong, Wuxi, and Changzhou. Soil was mainly brown and cinnamon, with saline–alkaline soils present in parts of western Xuzhou and Suqian and along the coastal zones of Lianyungang and Nantong. The relatively low importance value and biomass suggest that *S. canadensis* was present in these areas but had not yet developed strong community dominance (Table 1).

#### 2.1.3. Accompanying Species

**Figure 3:**
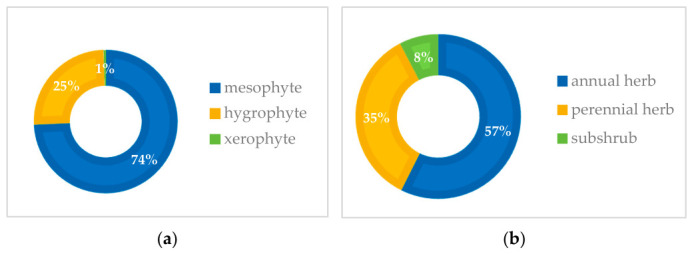
Composition of ecological moisture types and life forms of accompanying plant species in *S. canadensis*-invaded communities in Jiangsu Province. (**a**) Ecological moisture types. (**b**) Life forms.

The vegetation survey recorded 183 accompanying plant species from *S. canadensis*-invaded communities across Jiangsu Province, representing 125 genera and 43 families. Asteraceae, Poaceae, and Fabaceae were the most species-rich families, comprising 20 genera and 38 species, 26 genera and 36 species, and 10 genera and 13 species, respectively. According to the classification criteria described in the Methods, annual and biennial herbs were the dominant life-form category, with 105 species accounting for 57.38% of all recorded accompanying species, followed by perennial herbs (34.97%), whereas subshrubs represented only 7.65%. Regarding ecological moisture types, mesophytes comprised 74.31% of the accompanying species, followed by hygrophytes (25.14%), while xerophytes accounted for only 0.55% (Figure 3).

**Figure 4:**
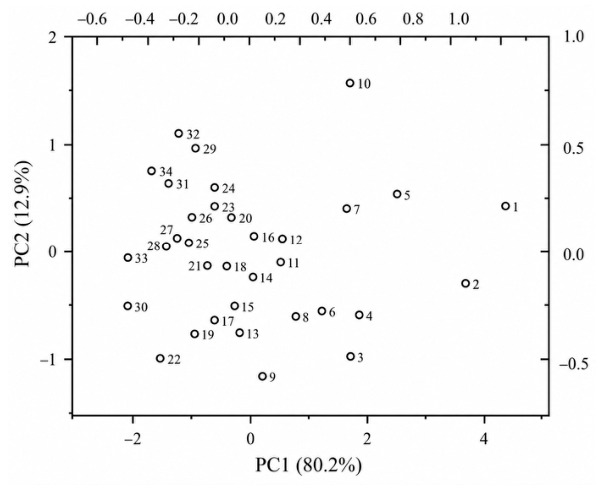
PCA ordination of dominant accompanying species associated with *S. canadensis*. 1 *S. canadensis*, 2 *S. viridis*, 3 *E. canadensis*, 4 *H. scandens*, 5 *D. sanguinalis*, 6 *S. subulatum*, 7 *S. faberi*, 8 *G. soja*, 9 *C. japonica*, 10 *C. dactylon*, 11 *P. australis*, 12 *A. lavandulifolia*, 13 *C. rostellatum*, 14 *V. radiata*, 15 *Lactuca indica* L., 16 *B. papyrifera*, 17 *P. foetida*, 18 *E. sumatrensis*, 19 *S. oleraceus*, 20 *M. alba*, 21 *I. purpurea*, 22 *G. spurium*, 23 *A. philoxeroides*, 24 *Echinochloa* spp., 25 *Sonchus brachyotus* DC., 26 *Achyranthes aspera* L., 27 *Acalypha australis* L., 28 *Cirsium arvense* var. *integrifolium* Wimm. & Grab., 29 *Rubus hirsutus* Thunb., 30 *I. triloba*, 31 *Artemisia lactiflora* Wall. ex DC., 32 *Euphorbia esula* L., 33 *E. annuus*, 34 *I. lacunosa*.

To visualize variation in dominant accompanying species, PCA was applied to the dominant species cover matrix (Figure 4). Formal tests of differences in accompanying-species composition among invasion-intensity groups are presented in Section 2.3.

*Setaria* spp. and *Digitaria sanguinalis* were common herbaceous species in disturbed habitats. Several species, including *Humulus scandens*, *Ipomoea purpurea* (L.) Roth, *Calystegia hederacea* Wall., *Cynanchum rostellatum* (Turcz.) Liede & Khanum, *Causonis japonica*, *Vigna radiata* (L.) R. Wilczek, *Ipomoea lacunosa* L., *Paederia foetida* L., *Ipomoea triloba* L., and *Galium spurium* L., showed climbing, twining, or vine-like growth forms. In addition, *Erigeron canadensis*, *Symphyotrichum subulatum*, *Alternanthera philoxeroides* (Mart.) Griseb., *Erigeron annuus* (L.) Pers., *Erigeron sumatrensis* Retz., and *Sonchus oleraceus* L. were recorded as alien, naturalized, or invasive taxa according to the criteria described in the Methods. Woody or perennial herbaceous components, such as *Broussonetia papyrifera* (L.) L’Hér. ex Vent., *Ligustrum lucidum* W.T. Aiton, *Morus alba* L., *Artemisia* spp., and *Melia azedarach* L., were also present in the invaded communities. The cover values of the main accompanying species across the three invasion-intensity groups are summarized in Table 2.

Overall, the accompanying flora of *S. canadensis*-invaded communities in Jiangsu Province was characterized by herbaceous dominance, a high proportion of mesophytes, and the coexistence of species with different growth forms and invasion statuses. This species inventory provides a baseline description of the plant assemblages associated with *S. canadensis* invasion, while the multivariate differences in accompanying-species composition among invasion-intensity groups are examined in Section 2.3.

#### 2.1.4. Alien and Invasive Taxa Within the Accompanying Flora

A total of 43 alien, naturalized, or invasive plant species were identified within the accompanying flora, belonging to 26 genera and 11 families. The most species-rich genera were *Ipomoea*, *Erigeron*, *Veronica*, and *Bidens*. Among these taxa, 58% were native to the Americas, including several representative alien, naturalized, or invasive taxa such as *A. philoxeroides*, *Amaranthus retroflexus* L., *Vicia sativa* L., *Veronica persica* Poir., *Veronica polita* Fr., *Euphorbia maculata* L., *I. triloba*, *S. subulatum*, *E. annuus*, *E. canadensis*, *E. sumatrensis*, and *Bidens frondosa* L. These taxa suggest that *S. canadensis*-invaded communities often contain multiple non-native or invasion-associated plant species, particularly in disturbed habitats and along anthropogenic dispersal pathways.

#### 2.1.5. Habitat Groups Associated with *S. canadensis* Occurrence

Based on the habitat classification criteria described in the Methods [28], the surveyed sites were assigned to five habitat groups: dense road-network sites, urban disturbed sites, park/woodland/orchard sites, agricultural sites, and riparian or wetland-margin sites. Field surveys showed that *S. canadensis* was most frequently recorded in dense road-network sites (Habitat Group I; 46.06% of the surveyed sites), followed by urban disturbed sites (Habitat Group II; 34.55% of the surveyed sites), particularly in areas undergoing land-use transformation, such as farmland converted to construction land or abandoned land converted to residential areas. These two habitat groups were dominated by annual and biennial herbs. *S. viridis*, *E. canadensis*, *H. scandens*, *D. sanguinalis*, and *S. subulatum* were frequently recorded. Vegetation in these habitats consisted mainly of pioneer or disturbance-tolerant species.

In parks, woodlands, and orchards (Habitat Group III; 16.97% of the surveyed sites), woody plants and perennial herbs constituted a larger proportion of the plant community. Climbing species such as *C. rostellatum* were commonly observed. Compared with the first two habitat groups, these sites had a relatively more complex vegetation structure, although vegetation gaps, edge effects, and human-mediated dispersal may still create opportunities for local establishment.

In agricultural land (Habitat Group IV; 0.60% of the surveyed sites), *S. canadensis* occurrence was rare, and the plant community was relatively simple, containing few accompanying species, most of which were crop-associated or common arable weeds such as *Echinochloa* spp. The agricultural record represented a single sampled site and was therefore presented descriptively, without inclusion in habitat-level statistical comparisons.

Along riverbanks, dikes, and canals (Habitat Group V; 1.82% of the surveyed sites), accompanying vegetation was dominated by species associated with relatively high moisture availability and linear dispersal corridors. Wetland or moisture-associated plants such as *P. australis* and *S. subulatum* were commonly observed. These habitats are characterized by abundant moisture and open dispersal pathways. Although *S. canadensis* is not a typical wetland species, it was recorded along wetland margins (Table 3). Together, these observations indicate that riparian and wetland-margin habitats may provide local establishment opportunities for *S. canadensis* along linear dispersal corridors, although this habitat group represented only a small proportion of the surveyed sites.

Overall, *S. canadensis* occurred widely across Jiangsu Province, but its population dominance, accompanying flora, and habitat associations varied among regions and invasion-intensity groups.

### 2.2. Factors Associated with the Distribution Pattern of S. canadensis in Jiangsu Province

**Figure 5:**
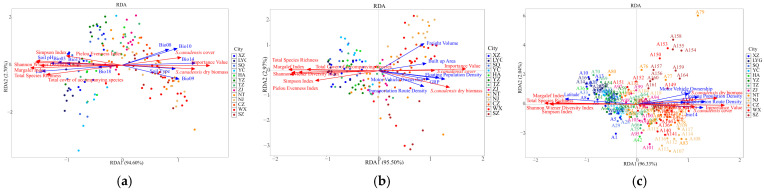
Redundancy analysis (RDA) showing the associations between *S. canadensis* invasion-related variables, accompanying plant diversity indices, and environmental or anthropogenic variables in Jiangsu Province, China. (**a**) RDA based on environmental variables; (**b**) RDA based on anthropogenic variables; (**c**) comprehensive RDA including selected environmental and anthropogenic variables. The environmental variables in Figure 5a include latitude, Bio03, Bio08, Bio09, Bio10, Bio13, Bio14, Bio18, soil type, and soil pH. The anthropogenic variables in Figure 5b include transportation route density, GDP, freight volume, floating population density, built-up area, and motor vehicle ownership. The variables in (**c**) include latitude, Bio14, floating population density, GDP, transportation route density, and motor vehicle ownership. Red arrows represent the response variables, including the *S. canadensis* invasion-related variables and accompanying-plant diversity indices, whereas blue arrows represent the environmental or anthropogenic explanatory variables. Arrow direction indicates the direction of association, and arrow length reflects the relative strength of the association. Hierarchical partitioning results with bootstrap-based 95% confidence intervals and VIF values are provided in Supplementary Table S2 and Table S3, respectively.

RDA (Figure 5) was used to examine the associations between selected environmental and anthropogenic variables and field-measured *S. canadensis* invasion indicators and accompanying-plant community variables in Jiangsu Province. All significance tests for the RDA models were permutation-based; because 999 permutations were used, *p* = 0.001 represents the lowest reportable permutation-based *p* value.

In the environmental RDA, the model explained 49.28% of the total variation and was statistically significant (R^2^ = 0.4928, adjusted R^2^ = 0.4599; F = 14.963, *p* = 0.001), with RDA1 and RDA2 explaining 94.60% and 2.70% of the constrained variation, respectively. Hierarchical partitioning showed that latitude had the highest relative contribution (21.71%), followed by soil pH (17.73%), precipitation of the driest month (Bio14) (15.57%), isothermality (Bio03) (11.17%), mean temperature of the driest quarter (Bio09) (7.88%), mean temperature of the warmest quarter (Bio10) (7.03%), soil type (6.98%), precipitation of the wettest month (Bio13) (6.60%), mean temperature of the wettest quarter (Bio08) (4.48%), and precipitation of the warmest quarter (Bio18) (0.85%). These results suggest that latitude, local soil conditions, and precipitation- or temperature-related climatic variables were closely associated with the observed invasion-related and community variables.

For the anthropogenic RDA, the model explained 54.81% of the total variation and was statistically significant (R^2^ = 0.5481, adjusted R^2^ = 0.5309; F = 31.935, *p* = 0.001), with RDA1 and RDA2 explaining 95.50% and 2.97% of the constrained variation, respectively. Among the anthropogenic variables, floating population density showed the highest relative contribution (22.25%), followed by GDP (19.33%), transportation route density (18.17%), freight volume (15.19%), motor vehicle ownership (12.76%), and built-up area (12.30%). These results suggest that population mobility, regional economic activity, transportation conditions, freight-related activity, and urbanization were the main anthropogenic correlates of the observed invasion-related and community variables in the fitted RDA model.

In the comprehensive RDA based on selected environmental and anthropogenic variables, the model explained 50.60% of the total variation and was statistically significant (R^2^ = 0.5060, adjusted R^2^ = 0.4873; F = 26.9761, *p* = 0.001), with RDA1 and RDA2 explaining 96.33% and 2.04% of the constrained variation, respectively. Hierarchical partitioning showed that latitude had the highest relative contribution (23.66%), followed by floating population density (18.47%), GDP (17.97%), precipitation of the driest month (Bio14) (17.16%), motor vehicle ownership (12.63%), and transportation route density (10.10%). Bootstrap-based 95% confidence intervals for all hierarchical partitioning percentages are provided in Supplementary Table S2. Overall, these results suggest that both environmental gradients and anthropogenic gradients were associated with the observed distribution pattern of *S. canadensis* in Jiangsu Province. Latitude may represent a broad geographic gradient, whereas anthropogenic gradients, especially population mobility and transportation route density and motor vehicle ownership, may help explain field occurrence patterns that are not fully captured by climatic gradients alone, particularly in northern Jiangsu. VIF diagnostics for the explanatory variables retained in each RDA model are provided in Supplementary Table S3.

### 2.3. Plant Diversity and Accompanying-Species Composition Along the S. canadensis Invasion-Intensity Gradient

Accompanying-plant community and diversity indices differed clearly among the three cluster-defined *S. canadensis* invasion-intensity groups (Table 4). Compared with the overall mean across all invaded sites, Group A showed significantly lower values for all measured community and diversity indices. The most substantial decreases occurred in the number of individuals of accompanying species after excluding *S. canadensis* (88.09% lower), total cover of accompanying species (72.04% lower), abundance of accompanying species (65.85% lower), and the Margalef index (49.15% lower). Other indices, including the Shannon–Wiener diversity index (46.91% lower), average height of accompanying species (44.15% lower), Simpson index (41.67% lower), total species richness (35.67% lower), and Pielou’s evenness index (30.43% lower), were also markedly reduced in Group A, indicating that the cluster-defined severe-invasion group was characterized by substantially lower accompanying-plant cover, abundance, richness, and diversity within the invaded-site dataset.

In Group B, several indices were higher than the overall means across invaded sites, including average height of accompanying species (40.58% higher), Pielou’s evenness index (24.64% higher), abundance of accompanying species (17.07% higher), Simpson index (14.58% higher), and Shannon–Wiener diversity index (12.35% higher). By contrast, total cover of accompanying species (31.69% lower) and the number of individuals of accompanying species after excluding *S. canadensis* (44.08% lower) remained below the overall means, while total species richness (0.67% lower) and the Margalef index (1.69% lower) were close to the overall means, indicating that Group B occupied an intermediate position along the invasion-intensity gradient.

In Group C, most diversity-related indices were higher than the overall means across invaded sites. Notably, abundance of accompanying species (68.29% higher), the Margalef index (61.02% higher), Shannon–Wiener diversity index (49.38% higher), total species richness (45.33% higher), Simpson index (41.67% higher), and Pielou’s evenness index (23.19% higher) showed substantial increases. Meanwhile, total cover, number of individuals, and average height of accompanying species were comparable to the overall means. These patterns indicate that the light-invasion group retained relatively higher accompanying-plant diversity within the invaded-site dataset (Table 4).

To test whether accompanying-species composition differed among the three cluster-defined invasion-intensity groups, PERMANOVA was performed using a Bray–Curtis dissimilarity matrix based on accompanying-species cover data. *S. canadensis* was excluded from the species matrix because the invasion-intensity groups were defined using its population traits. PERMANOVA detected a significant overall difference in accompanying-species composition among the three groups (Pseudo-F = 2.97, R^2^ = 0.035, *p* < 0.001; Table 5).

A multivariate dispersion test was then conducted to examine whether within-group variability differed among the three invasion-intensity groups. The result was also significant (F = 13.39, *p* < 0.001; Table 5), indicating that the multivariate pattern detected by PERMANOVA reflected both differences in group centroids and differences in within-group dispersion. Therefore, the PERMANOVA result should be interpreted as evidence of a detectable difference in accompanying-species composition among invasion-intensity groups while also considering the observed differences in within-group variability.

The occurrence of sites without accompanying species was further examined. The proportion of sites without accompanying species differed significantly among the three groups (Fisher’s exact test, *p* = 0.011; Table 5). In Group A, 7 of 74 sites had no accompanying species recorded after excluding *S. canadensis*. In contrast, at least one accompanying species was recorded in every site in Groups B and C.

Finally, a sensitivity PERMANOVA was performed after excluding sites without accompanying species. In this reduced dataset (*n* = 158), the PERMANOVA result remained significant (Pseudo-F = 2.64, R^2^ = 0.033, *p* < 0.001; Table 5). This result indicates that the difference in accompanying-species composition among invasion-intensity groups was not driven solely by sites without accompanying species. Overall, the species-level analysis showed that accompanying-species composition differed significantly along the *S. canadensis* invasion-intensity gradient within the invaded-site dataset, although the proportion of explained variation was limited. The consistency between the full and reduced datasets further supports a detectable compositional difference along the invasion-intensity gradient.

### 2.4. Climatic Habitat Suitability and Field-Observed Invasion Intensity of S. canadensis

**Figure 6:**
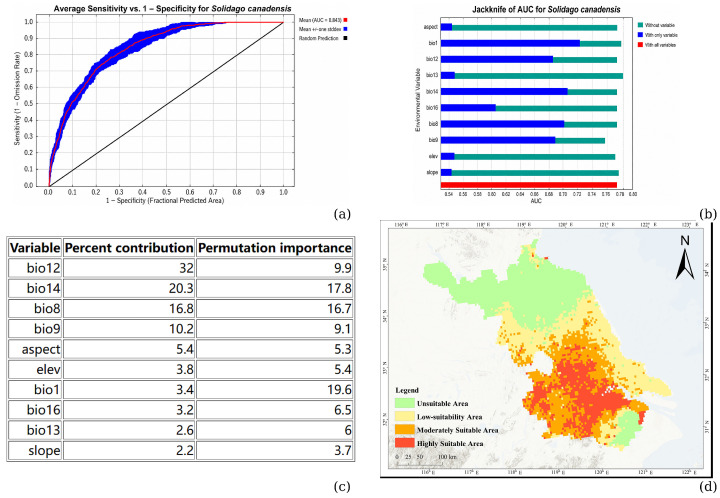
MaxEnt-based prediction of climatic habitat suitability for *S. canadensis* in Jiangsu Province. Note: (**a**) Receiver operating characteristic (ROC) curve of the MaxEnt model; (**b**,**c**) Relative contributions of environmental variables and jackknife test results; (**d**) Predicted habitat suitability overlaid with field survey records of *S. canadensis* in Jiangsu Province. The model was parameterized as described in the Methods; mean training AUC = 0.843 and mean test AUC = 0.787 ± 0.036.

The model’s predictive performance was evaluated using the receiver operating characteristic (ROC) curve and its corresponding area under the curve (AUC) value. The model had a mean training AUC of 0.843 (Figure 6), indicating acceptable discriminatory ability for a presence-only climatic suitability model [11]. The jackknife test, together with the variable contribution results (Figure 6b,c), suggested that the predicted climatic habitat suitability of *S. canadensis* was mainly associated with Bio12, Bio14, Bio8, and Bio9, corresponding to annual precipitation, precipitation of the driest month, mean temperature of the wettest quarter, and mean temperature of the driest quarter, respectively. These results indicate that hydrothermal conditions were important climatic correlates of predicted habitat suitability for *S. canadensis*. The predicted habitat suitability map (Figure 6d) showed that southern and central Jiangsu had relatively higher predicted climatic suitability, whereas northern Jiangsu was mainly predicted as unsuitable or low-suitability habitat.

To compare the climate-based suitability prediction with field-observed invasion intensity, the 165 field survey sites were overlaid with the MaxEnt suitability classes. This cross-tabulation was used as a descriptive comparison between model-based climatic suitability classes and field-observed invasion-intensity groups.

At the provincial scale, 82 field sites were in high-suitability areas, including 53 severe-invasion sites, 20 moderate-invasion sites, and 9 light-invasion sites (Table 6). In contrast, 67 sites were in areas predicted as unsuitable or low-suitability, including 12 severe-invasion sites, 35 moderate-invasion sites, and 20 light-invasion sites. These results indicate that the MaxEnt model captured the broad climatic suitability pattern of *S. canadensis*, but climatic suitability alone did not fully explain field-observed occurrence or local invasion intensity.

The discrepancy between predicted climatic suitability and field observations was more evident in northern Jiangsu. Among the 51 northern Jiangsu sites, 46 sites were in areas predicted as unsuitable or low suitability by the MaxEnt model. Nevertheless, these sites included one severe-invasion site and 25 moderate-invasion sites (Table 6). This pattern suggests that factors not included in the climate-based MaxEnt model, such as human-mediated dispersal, transportation corridors, disturbed habitats, and local microhabitat conditions, may contribute to field occurrence and local invasion intensity in northern Jiangsu.

Overall, the MaxEnt model identified southern and central Jiangsu as the main climatically suitable areas for *S. canadensis*. However, field observations confirmed the occurrence of *S. canadensis* populations in northern Jiangsu, including sites with moderate or severe invasion intensity, despite generally lower predicted climatic suitability in this region. This partial mismatch suggests that northern Jiangsu should not be regarded as unsuitable based solely on climate-based MaxEnt predictions. Instead, northern Jiangsu should be considered an area requiring continued monitoring of field occurrence and local establishment, particularly in disturbed habitats and along transportation corridors where local establishment may occur despite lower climatic suitability.

## 3. Discussion

### 3.1. Distribution of S. canadensis in Jiangsu Province and Its Associated Factors

The field observations showed that the invasion intensity of *S. canadensis* in Jiangsu Province followed a clear south-to-north decreasing gradient, with severe and moderate invasion concentrated in southern and central Jiangsu. This spatial gradient is consistent with the species’ early introduction and establishment history in southern Jiangsu and the broader Yangtze River Delta region. In the early 20th century, *S. canadensis* was introduced as a garden ornamental along the Shanghai–southern Jiangsu–northern Zhejiang urban corridor, which likely provided important initial introduction sites and subsequent dispersal opportunities across the region [2,29,30]. The concentration of high-intensity invasion in southern Jiangsu may therefore reflect the combined effects of earlier residence time, intensive land-use change, dense transportation networks, and frequent human-mediated propagule dispersal.

Ecologically, latitude is generally associated with climatic gradients and regional differences in vegetation and ecosystem conditions [31,32]. In this study, latitude showed the highest relative contribution in the environmental RDA (21.71%) and remained the most important variable in the comprehensive RDA (23.66%). However, latitude should be interpreted as an integrative geographic gradient rather than a direct causal factor. In Jiangsu, the latitudinal gradient corresponds broadly to changes in hydrothermal conditions, vegetation zones, land-use intensity, and urbanization. Qiang et al. [33] documented sharp differences in weed communities across the Huai River near 33° N, where subtropical and temperate vegetation zones meet. The variation in accompanying-plant diversity observed in this study may therefore partly reflect this regional ecological transition. Soil pH also showed a relatively high contribution in the environmental RDA (17.73%), suggesting that local edaphic conditions may be associated with the observed invasion-related and community variables. Precipitation of the driest month (Bio14) was also an important precipitation-related climatic variable, with a relative contribution of 15.57% in the environmental RDA and 17.16% in the comprehensive RDA. In the MaxEnt model, hydrothermal variables, including Bio12 and Bio14, were also important correlates of predicted climatic habitat suitability. Together, these results indicate that water availability, especially during relatively dry periods, may be an important climatic correlate of both field-observed invasion intensity and predicted habitat suitability of *S. canadensis* in Jiangsu Province.

The comparison between MaxEnt-predicted climatic suitability and field observations further refines this interpretation. The MaxEnt model captured the broad climatic suitability pattern, but field surveys showed that *S. canadensis* also occurred in northern Jiangsu, including sites with moderate or severe invasion intensity, despite generally lower predicted climatic suitability in this region. Similar issues have been reported in recent species distribution modeling studies, where field validation and observed occurrence records were needed to refine model-based risk interpretation [34,35,36]. In the present study, the discrepancy in northern Jiangsu suggests that local establishment may also depend on non-climatic processes. Transportation corridors, disturbed habitats, propagule pressure, and local microhabitat conditions may allow *S. canadensis* to occur in areas where climatic suitability is predicted to be low. This contrast highlights the complementary roles of climate-based suitability modeling and field surveys: MaxEnt captured broad climatic constraints, whereas field observations revealed local establishment in disturbed or human-modified habitats. Therefore, these results support continued monitoring in northern Jiangsu.

From an anthropogenic perspective, floating population density, GDP, and transportation route density were the main anthropogenic variables associated with the observed invasion and community patterns of *S. canadensis*. Hierarchical partitioning showed that floating population density had the highest relative contribution in the anthropogenic RDA (22.25%), followed by GDP (19.33%) and transportation route density (18.17%). Freight volume also showed a relatively high contribution (15.19%), suggesting that logistics-related activities may be associated with observed invasion and community patterns. These variables may represent processes such as propagule pressure, habitat disturbance, contaminated soil movement, and repeated introduction along transportation corridors. These socioeconomic indicators jointly reflected broader gradients of regional development, urbanization, transportation activity, and human mobility, which may collectively influence the observed distribution pattern of *S. canadensis*. Studies of eastern ports and transport systems suggest that ports and major corridors may act as primary introduction points and secondary dispersal pathways, consistent with the formation of high-risk zones in southern and central Jiangsu [15]. Genetic evidence further supports this possibility, revealing multiple introductions and the coexistence of several genetic groups in eastern China and the Yangtze River Delta [37]. Additionally, research in coastal and riparian habitats comparable to Jiangsu’s coastal zone has documented the establishment of *S. canadensis* and associated soil-property changes, suggesting that these habitats still require close monitoring even where local population size or spatial expansion remains limited [19]. Overall, these findings suggest that the invasion pattern of *S. canadensis* in Jiangsu is associated not only with climatic gradients but also with human-mediated dispersal and habitat disturbance.

Global warming may further alter the potential distribution and invasion risk of *S. canadensis* by increasing suitability in regions experiencing warmer cold seasons and altered dry-season moisture conditions [13]. Previous studies have suggested that warming and nitrogen enrichment can enhance the performance and competitiveness of *S. canadensis* [16]. The importance of Bio14 in both the RDA and MaxEnt analyses suggests that dry-season precipitation may be related to the potential distribution of *S. canadensis*. Under future climate warming, changes in cold-season temperature and dry-season moisture may alter latitudinal constraints and influence the south–north gradient in invasion intensity [32]. Taken together, these results suggest that invasion risk under future climate and disturbance scenarios deserves continued monitoring, particularly in climatically transitional areas such as northern Jiangsu.

### 3.2. Community Patterns Along the S. canadensis Invasion-Intensity Gradient

Field observations in this study showed clear differences in accompanying-plant community structure and diversity among sites with different invasion intensities of *S. canadensis*. These community patterns are consistent with previous studies showing that *S. canadensis*-invaded communities often differ in understory plant diversity, community stability, and soil properties [19]. Together, these findings suggest that variation in community structure and diversity was detectably associated with the *S. canadensis* invasion-intensity gradient in the surveyed sites.

Plant diversity is closely related to community stability and resistance to invasion [38,39,40,41]. In the present study, sites with high invasion intensity were generally associated with lower diversity indices and lower abundance of accompanying species, especially in southern Jiangsu. Previous studies have shown that, in warmer regions, *S. canadensis* may exhibit stronger competitive ability and may form monodominant stands that suppress native plant species [16,42,43,44]. In lower-latitude regions, invasive species and their co-occurring species may converge toward similar ecological niches, potentially leading to homogenization and reduced diversity [38,41]. This may help explain why heavily invaded sites in southern Jiangsu showed simpler community structure and lower accompanying species abundance. Such declines in diversity may increase community sensitivity to disturbance and climate change, as suggested by previous studies [45,46]. Previous research has also reported that *S. canadensis* invasion is associated with changes in soil structure, vegetation distribution, and microclimatic conditions in wetlands and shelterbelts, which may further affect native plant performance and community recovery [43,47,48]. Taken together, these studies provide plausible ecological context for the lower accompanying-plant diversity and simpler community structure observed in heavily invaded sites.

The community patterns observed in this study may be related to several ecological processes reported in previous studies. *S. canadensis* is known to have strong resource acquisition capacity, particularly for nitrogen and phosphorus, which may alter local nutrient availability, moisture retention, and nutrient cycling [16,49,50]. Recent evidence further suggests that soil phosphorus forms and arbuscular mycorrhizal fungi can influence the growth advantage of *S. canadensis*, indicating that belowground nutrient pathways may contribute to its invasion performance [50]. Such shifts in resource availability and belowground interactions may reduce the performance of species that are less tolerant of altered nutrient conditions or that depend on specific nutrient-rich microsites [20,51]. In addition, previous studies have shown that *S. canadensis* invasion can be associated with changes in soil chemistry and microbial community composition, which may further influence plant community composition and ecosystem resilience [16,41,52,53]. Recent research has also shown that litter leachates and root exudates of *S. canadensis* can modify soil physicochemical properties and bacterial communities, providing a possible pathway for plant–soil feedback during invasion [22]. Therefore, resource competition, soil-mediated feedback, and allelopathic interference may provide plausible explanations for the reduced diversity and simplified community structure observed in heavily invaded sites. These mechanisms are consistent with the field patterns observed here, but their relative importance could be further clarified by studies that combine vegetation surveys with direct measurements of soil microbial communities, allelopathic effects, and resource uptake [45].

As globalization accelerates, the global spread of invasive plants is increasing, and the concept of “invasion debt” highlights the difficulty of responding effectively once invasions have become widespread. The relative importance of driving factors may also vary across invasion stages [54]. Recent research on alien plant invasion debt has further shown that future invasion risk can be shaped by habitat conditions, human disturbance, and dispersal pathways [55]. Within this broader context, northern Jiangsu may face rising invasion pressure. Limiting the adaptive spread of invasive plants while enhancing ecosystem resistance during urbanization remains a long-term challenge [56]. Therefore, future monitoring should focus not only on the cover and distribution of *S. canadensis*, but also on changes in accompanying-species composition, co-occurring alien plants, and habitat disturbance.

### 3.3. Control Strategies for S. canadensis

Marked differences in natural conditions and socioeconomic development between southern and northern Jiangsu, combined with habitat-specific variation in *S. canadensis* occurrence, may contribute to regional differences in invasion risk. Accordingly, control measures should be adapted to site-specific conditions within a management framework emphasizing zoning management, source control, and integrated management [15,57]. The field survey in this study identified priority regions and habitat types for management, while the specific technical measures discussed below are proposed by integrating these field-observed patterns with previous studies on habitat-based invasion risk, invasive plant monitoring, mechanical control, competitive vegetation restoration, and ecological restoration [57,58,59,60,61,62,63,64,65]. Together, this evidence provides a basis for practical management options that require local testing before broader application.

1. Key habitat and high-risk area management.

In high-risk habitats, management should prioritize transportation corridors and highly disturbed urban spaces such as urban junctions, construction sites, and abandoned land [57]. An integrated monitoring–removal–restoration approach may be appropriate. Recent advances in UAV-based remote sensing and machine-learning-assisted mapping may provide useful supplementary tools for future monitoring of invasive plant patches, but their applicability in Jiangsu should be validated under local habitat and phenological conditions [59,60,61,62]. In Jiangsu, these tools could be explored to help identify large or scattered patches along transportation corridors, abandoned land, and other highly disturbed habitats. When combined with ground surveys and phenological information, such monitoring could help locate priority patches, track post-removal vegetation recovery, and evaluate reinvasion risk. In transportation corridors subject to intense disturbance, removal should be combined with sustained zone-based management to reduce the risk of long-term dominance. Restoration planning should incorporate assessment of existing vegetation structure, post-removal recovery capacity, and long-term monitoring needs. Repeated removal before flowering, combined with restoration of locally suitable native or locally established competitive species, may help reduce seed input, available establishment space, and subsequent reinvasion pressure [63,64,65]. In addition, recently developed biologically based control approaches for *S. canadensis* may provide useful research directions, but their feasibility and ecological safety should be evaluated under local conditions before practical application [66]. In riparian and wetland-margin habitats, management should focus on linear dispersal pathways and the maintenance of existing vegetation cover. Where appropriate, locally occurring wetland plants such as *Phragmites australis* may be considered as habitat-specific restoration candidates, but their effectiveness should be evaluated under local habitat conditions. Previous studies on hydrochorous seed dispersal suggest that water pathways can influence propagule movement in riparian systems [58].

2. Regional zoning and tiered control.

Based on the observed invasion-intensity patterns and habitat occurrence characteristics, we suggest three-tiered control categories for the management of *S. canadensis* in Jiangsu Province.

(1) Severe invasion zone (Group A): In areas with dense populations and developed transportation networks, management should prioritize potential dispersal pathways associated with transportation, logistics, land-use change, and plant material movement [15,57]. Where feasible, rhizome removal, careful disposal of plant residues, and mechanical control may be combined with repeated monitoring and long-term restoration. Post-treatment restoration should aim to establish stable communities dominated by locally suitable native perennial herbs, shrubs, or woody species to increase vegetation cover and reduce open niches, in line with the concepts of ecological restoration and community resistance after invasive plant management [64].
(2) Moderate invasion zone (Group B): Physical removal and vegetation restoration should be combined, with clearing or mowing during the seedling stage in spring and before flowering in autumn. Planting suitable native or locally established competitive species may help rebuild competitive barriers. For *Solidago* spp., repeated mechanical removal combined with ecological restoration has been shown to reduce regenerative capacity and cover [65].
(3) Light invasion zone (Group C): For lightly invaded areas, management should focus on maintaining existing vegetation cover and restoring ecological barriers, particularly in wetland and riparian-margin communities. Restoration planning in these areas should prioritize locally suitable resident vegetation. This strategy is broadly consistent with the view that higher local diversity and the prior establishment of resident plant species with strong competitive ability can increase community resistance to invasion [67,68,69,70].

3. Restoration implications of vegetation structure in *S. canadensis*-invaded communities.

Human disturbance can interact with community recovery processes by maintaining open niches, thereby prolonging invasive dominance [69].

Field surveys in Group A areas, such as Nanjing, Wuxi, and Changzhou, showed that some *S. canadensis*-invaded communities contained woody or climbing plant components in addition to herbaceous species. Communities in these areas appeared to differ from strongly invaded annual-herb-dominated stands in terms of vegetation structure and species composition. Although the present survey cannot reconstruct long-term successional trajectories, the occurrence of woody and climbing components suggests that existing vegetation structure and site disturbance history should be considered when designing restoration strategies. In sites with persistent disturbance, *S. canadensis* dominance may be prolonged where repeated disturbance maintains open niches and facilitates invasive persistence. Therefore, reducing unnecessary disturbance and promoting vegetation recovery may help improve community resistance and reduce reinvasion risk [70]. In the present survey, climbing herbaceous vines, fast-growing woody species such as *Broussonetia papyrifera*, and perennial rhizomatous plants such as *Miscanthus sacchariflorus* were observed in some invaded communities and may provide field-based clues for selecting locally suitable functional groups in restoration planning. However, their practical restoration value should be further evaluated through local restoration trials or long-term monitoring before practical application.

## 4. Materials and Methods

### 4.1. Field Survey

**Figure 7:**
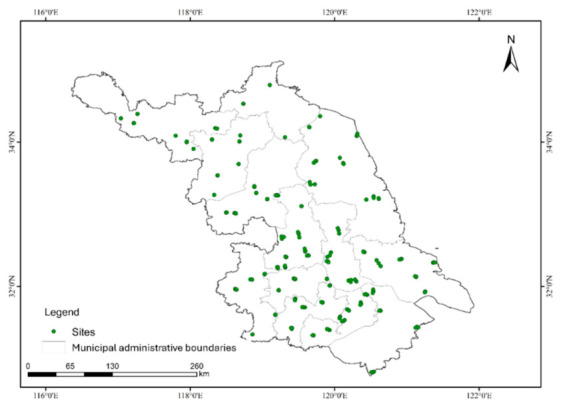
Locations of field survey sites of *S. canadensis* in Jiangsu Province.

Field surveys were conducted in autumn 2022. A total of 165 sampling sites were established in Jiangsu Province (Figure 7). Sites were selected according to (1) previous occurrence records of *S. canadensis*, (2) field reconnaissance confirming its current presence or likely occurrence, and (3) habitat representativeness across the major invaded habitat types of the province. To reduce spatial bias, only one representative site was selected from each continuous infestation patch whenever possible, and sites were distributed across different cities and habitat types [71].

During the field survey, habitat type was recorded for each sampling site according to predefined field criteria, including dominant land-use type, major disturbance source, moisture condition, and vegetation structure. The habitat classification followed previous field investigations of *S. canadensis* in Jiangsu Province and habitat-classification frameworks used in alien *Solidago* studies [9,28,72]. Previous surveys reported that *S. canadensis* in Jiangsu Province mainly occurred along roadsides, riverbanks, development zones, and coastal embankments [28]. Similar habitat-based classification has also been used in studies of alien *Solidago* taxa and broader alien plant invasions across different habitat types [73]. Sites along highways, sidewalks, rural roads, railway crossings, roadside slope-protection zones, road embankments, and interchange zones were recorded as dense road-network sites. Sites in urban junctions, residential zones, construction sites, abandoned land, or land-use transition zones with obvious anthropogenic disturbance were recorded as urban disturbed sites. Sites in parks, woodlands, orchards, or green spaces with relatively more woody plants and perennial herbs were classified as park/woodland/orchard sites. Sites in cropland, field margins, or areas under regular agricultural management were recorded as agricultural sites. Sites along riverbanks, dikes, canals, water edges, or wetland margins were classified as riparian or wetland-margin sites [9,28,72,73].

At each site, five 1 m × 1 m quadrats were arranged along a diagonal transect, with intervals of more than 10 m between adjacent quadrats, following commonly used herbaceous vegetation survey and terrestrial vegetation monitoring methods [74,75,76,77]. The five spatially separated quadrats at each site were averaged to obtain site-level values for subsequent analyses. This design was used to ensure sampling consistency and comparability across 165 province-wide sites. Longitude, latitude, and elevation were recorded using a Garmin eTrex 32x handheld GPS receiver (Garmin International, Inc., Olathe, KS, USA).

Life forms, ecological moisture types, growth forms, and invasion status of accompanying species were determined according to documented species-level traits and authoritative floristic or invasion-related references. Life forms were classified as annual herbs, biennial herbs, perennial herbs, and subshrubs, based on standardized plant functional-trait and vegetation-description frameworks [78,79,80]. Ecological moisture types were classified as mesophytes, hygrophytes, and xerophytes according to species habitat preferences and field-recorded moisture conditions, with species-level information checked using *Flora of China*, the Chinese Virtual Herbarium, and related floristic references [81,82]. Growth forms, including herbaceous, woody, climbing, twining, or vine-like habits, were verified using *Flora of China*, the Chinese Virtual Herbarium, and related floristic references [81,82]. Invasion status was determined according to established concepts of alien, naturalized, and invasive plants, as well as Chinese invasive plant checklists or databases [83,84].

All quadrat-based community analyses were conducted at sites where *S. canadensis* was present. Because paired uninvaded reference plots matched by habitat type, disturbance intensity, soil conditions, and geographic location were not included in this study, comparisons of plant diversity among Groups A–C are interpreted as patterns of diversity variation along an invasion-intensity gradient within invaded communities.

Unidentified plants were photographed or collected and subsequently identified using CVH (https://www.cvh.ac.cn, accessed on 18 February 2026) and Flora of China resources (http://www.iplant.cn/frps, accessed on 18 February 2026). The alien, naturalized, or invasive status of each species was checked using the Chinese invasive plant database (http://www.iplant.cn/ias, accessed on 18 February 2026), Invasive Alien Plants of China [84], and Invasive Alien Organisms of China [85].

For each quadrat, plant height, cover, and biomass were recorded. Biomass samples were dried to constant weight in DHG-9240A forced-air drying oven (Shanghai Yiheng Scientific Instrument Co., Ltd., Shanghai, China). Soil type was recorded in the field, and soil pH was measured after sieving using a PHS-3C pH meter (Shanghai INESA Scientific Instrument Co., Ltd., Shanghai, China). Additional occurrence records for which quadrat establishment was not feasible were retained only as occurrence-only data, including species occurrence, approximate occurrence area, and GPS coordinates. These records were excluded from quadrat-based community analyses [86].

### 4.2. Species Occurrence Data for MaxEnt Modeling

Occurrence records of *S. canadensis* in Jiangsu Province were compiled from historical survey records, the National Specimen Information Infrastructure (NSII; https://www.nsii.org.cn/2017/home-en.php, accessed on 10 March 2026), the Chinese Virtual Herbarium (CVH, http://www.cvh.ac.cn, accessed on 10 March 2026), and field surveys conducted in autumn 2022. Base maps were obtained from the National Fundamental Geographic Information System (http://nfgis.nsdi.gov.cn, accessed on 10 March 2026). All records were checked for geographic accuracy and georeferenced when necessary, and duplicate, invalid, or ambiguous records were removed prior to analysis.

To reduce spatial sampling bias and spatial autocorrelation, occurrence records were spatially thinned at the environmental raster-cell scale. The ASCII environmental layers used for MaxEnt version 3.4.4 had a cell size of 0.0416667° (2.5 arc-min, approximately 4–5 km), and only one representative occurrence record was retained within each raster cell whenever multiple records occurred in the same cell [87]. After spatial thinning, 96 occurrence records were retained for the final MaxEnt model. These records were occurrence-only data and were used only to indicate the presence locations of *S. canadensis* for climatic habitat suitability modeling. Specifically, the 165 quadrat-survey sites were field sites where five 1 m × 1 m quadrats were established and vegetation variables were measured, whereas the 96 MaxEnt occurrence records were spatially thinned presence records at the environmental raster-cell scale.

### 4.3. Environmental Variables for MaxEnt Modeling

Environmental variables consisted of 19 bioclimatic factors and three terrain-related factors, namely elevation, slope, and aspect. The bioclimatic variables and elevation data were obtained from the WorldClim version 2.1 (https://www.worldclim.org/data/worldclim21.html, accessed on 10 March 2026), while slope and aspect were derived from the elevation layer. Detailed descriptions of these variables are provided in Table 7.

To reduce strong pairwise redundancy among environmental predictors used in MaxEnt, Pearson correlation analysis was conducted using IBM SPSS Statistics 27.0 (IBM Corp., Armonk, NY, USA). When two variables showed strong correlation (|r| > 0.8), one variable was selected and retained according to its ecological relevance and preliminary model performance. The final MaxEnt predictor set included aspect, elevation, slope, Bio1, Bio8, Bio9, Bio12, Bio13, Bio14, and Bio16. The relative contribution of the final variables to the predicted distribution of *S. canadensis* was then assessed [86,88].

Human disturbance and community structure variables were not included in the MaxEnt model. These variables were analyzed separately in subsequent statistical analyses. This separation was used to distinguish climate-based habitat suitability modeling from field-based analyses of invasion intensity, community structure, and anthropogenic disturbance.

### 4.4. Model Settings and Evaluation

MaxEnt version 3.4.4 was run for 10 bootstrap replicates, using 75% of the spatially thinned occurrence records for training and 25% for testing, with logistic output selected such that model predictions represented relative habitat suitability [86,88]. After spatial thinning, 96 occurrence records were retained for modeling, including 72 training records and 24 test records. The final model used 5545 background points. These background points were randomly sampled from the environmental raster layers within the Jiangsu Province study-area mask, excluding NoData cells.

To avoid overfitting and improve predictive performance, MaxEnt model parameters were optimized by comparing different feature-class combinations and regularization multipliers. Candidate feature-class combinations included L, LQ, H, LQH, LQHP, and LQHPT, and candidate regularization multipliers ranged from 0.5 to 4.0 at intervals of 0.5. The optimal parameter combination was selected based on the lowest AICc while also considering omission rate and validation AUC [89,90,91]. The parameter-optimization results indicated that the optimal combination was LQH with a regularization multiplier of 4.0 (AICc = 1590.63, delta AICc = 0).

The model’s predictive ability was evaluated using the receiver operating characteristic (ROC) curve and its corresponding area under the curve (AUC) value [86,88]. The model had a mean training AUC of 0.843 and a mean test AUC of 0.787 ± 0.036, indicating acceptable to reasonably good model discrimination under a presence-only modeling framework [88]. Therefore, MaxEnt outputs were used to identify broad-scale relative habitat suitability patterns and compare the relative importance of environmental predictors.

### 4.5. Habitat Suitability Classification

The logistic output of MaxEnt was converted to raster format in ArcGIS Desktop 10.8, and the final distribution suitability map for *S. canadensis* was derived through mask extraction. Predicted habitat suitability was classified into four levels: unsuitable (<0.185486), low suitability (0.185486–0.340092), moderate suitability (0.340092–0.473936), and high suitability (>0.473936) [87,88]. To compare MaxEnt-predicted habitat suitability with field-observed invasion intensity, the GPS coordinates of the 165 sampling sites were overlaid on the classified habitat-suitability raster in ArcGIS. The predicted suitability class of each site was extracted and matched with its cluster-defined invasion-intensity group, namely Group A, Group B, or Group C. A cross-tabulation was then used to summarize the correspondence between predicted suitability classes and observed invasion-intensity groups. The same procedure was also applied to the northern Jiangsu subset to examine regional mismatch between model prediction and field observations. This comparison was used as a descriptive evaluation of model–field consistency [34,35,36].

### 4.6. Anthropogenic Variables

Anthropogenic variables were obtained from the Jiangsu Statistical Yearbook 2024, published by the Jiangsu Provincial Bureau of Statistics [92], including transportation route density, GDP, freight volume, floating population density, built-up area, and motor vehicle ownership. Transportation route density was calculated as total transportation mileage divided by city area, and floating-population density was calculated as the reported floating population divided by city area. These variables were not used as predictors in MaxEnt but were included in subsequent multivariate analyses to assess their associations with the observed invasion-related and accompanying-community patterns of *S. canadensis*.

### 4.7. Community Variables and Statistical Analyses

Fifteen variables were used to characterize the accompanying-community structure, *S. canadensis* population attributes, and plant-diversity patterns within invaded plots. These variables included five accompanying-community variables: (1) total cover of other species; (2) accompanying species richness, defined as the total number of species other than *S. canadensis*; (3) average height of other species; (4) relative abundance of accompanying species, calculated as the proportion of accompanying-species individuals relative to the total number of plant individuals in each plot; and (5) total plant species richness.

The population-related variables of *S. canadensis* included: (6) average height of *S. canadensis*, measured from five randomly selected individuals in each plot; (7) cover of *S. canadensis*, visually estimated as the percentage cover of *S. canadensis* in each plot; (8) frequency of *S. canadensis*, defined as the number of occurrences of *S. canadensis* across the quadrat records; (9) total number of *S. canadensis* individuals; (10) importance value; and (11) *S. canadensis* dry biomass.

The importance value (IV) of *S. canadensis* was calculated as the means of relative cover, relative frequency, and relative density: IV = (relative cover + relative frequency + relative density)/3, where relative cover, relative frequency, and relative density represent the corresponding relative values of *S. canadensis* in each plot.

The diversity indices included: (12) Simpson index; (13) Shannon–Wiener diversity index, calculated as H′ = −Σ(P_i_ ln P_i_), where P_i_ is the proportion of individuals belonging to species i among all recorded individuals; (14) Pielou’s evenness index, calculated as J = H′/ln S, where S is the number of species in each plot; and (15) Margalef’s richness index, calculated as D_m_g = (S − 1)/ln N, where N represents the total number of plant individuals recorded in the plot [86]. For clonal species, density was recorded using visible aboveground shoots, ramets, tillers, or culms rooted within each quadrat as the operational counting unit [93,94,95].

Invasion-intensity groups were defined using hierarchical cluster analysis based on five Z-score-standardized field-measured variables of *S. canadensis*: cover, frequency, individual density, biomass, and height. Pairwise dissimilarity among the 165 sampling sites was calculated using Euclidean distance, and hierarchical clustering was performed using Ward’s linkage method. The resulting dendrogram was cut into three clusters, corresponding to severe, moderate, and light invasion groups. The three-cluster solution was selected based on the dendrogram structure, especially the marked increase in fusion distance before the final major cluster merges, and on ecological interpretability as low-, intermediate-, and high-dominance invasion groups. PCA was used only as an ordination method to visualize the separation among the cluster-defined groups [96].

Microsoft Excel 2016 was used for preliminary data management, IBM SPSS Statistics 27.0 was employed for statistical analysis, and figures were prepared in OriginPro 2023 [97]. Because the first-axis gradient length was less than 3, redundancy analysis (RDA), a linear ordination method, was applied. RDA was performed using R version 4.5.3 with the vegan package version 2.7-3 to examine the relationships between plot-level community attributes and environmental or anthropogenic variables [96,98]. All explanatory variables were standardized before analysis to reduce the influence of different units and scales. Variance inflation factors (VIFs) were used to assess multicollinearity among the explanatory variables retained in each RDA model, and the resulting VIF values are reported in Supplementary Table S3. The explanatory power of the RDA models was evaluated using R^2^, adjusted R^2^, and the percentages of constrained variation explained by RDA1 and RDA2. The significance of the overall RDA models, RDA axes, explanatory variables, and fitted explanatory-variable vectors was tested using 999 permutations. Because 999 permutations were used for the RDA tests, 0.001 was the lowest reportable permutation-based *p* value. Hierarchical partitioning was further conducted using the rdacca.hp package version 1.1-2 to quantify the relative contribution of each explanatory variable [99]. Bootstrap resampling was used to estimate 95% confidence intervals for the relative contributions obtained from hierarchical partitioning. Occurrence-only data were used for MaxEnt modeling, whereas quadrat-based data were used for community analyses and invasion intensity comparisons.

To test whether accompanying-species composition differed among the three cluster-defined invasion-intensity groups, a site-by-species matrix was constructed using the cover values of accompanying species. *S. canadensis* was excluded from this matrix because the invasion-intensity groups were defined using its population traits. Bray–Curtis dissimilarity was calculated based on the accompanying-species cover matrix, and PERMANOVA was performed to test for overall compositional differences among the three groups using 9999 permutations [100]. The PERMANOVA R^2^ value was reported as the effect size for compositional differences among invasion-intensity groups. Because PERMANOVA results may be affected by differences in multivariate dispersion among groups, a multivariate dispersion test was further conducted using the same Bray–Curtis dissimilarity matrix [101,102]. Because some expected frequencies were below 5, Fisher’s exact test was used to compare the proportion of sites without accompanying species among the three groups. To examine whether the PERMANOVA result was driven solely by sites without accompanying species, a sensitivity PERMANOVA was performed after excluding these sites. The PERMANOVA and multivariate dispersion analyses were conducted using the adonis2 and betadisper functions in the vegan version 2.7-3 [98]. Statistical significance was assessed at *p* < 0.05.

## 5. Conclusions

This province-wide survey provides a field-based assessment of the occurrence, invasion-intensity gradient, and community characteristics of *Solidago canadensis* in Jiangsu Province. The species was recorded across the province, but its occurrence and dominance were unevenly distributed, with higher invasion intensity and dominance mainly concentrated in southern Jiangsu and weaker dominance in northern and coastal areas. Hierarchical cluster analysis based on field-measured population traits separated the invaded sites into severe, moderate, and light invasion-intensity groups, indicating clear spatial heterogeneity in invasion status across the province.

Within the invaded-site dataset, severely invaded sites were generally associated with lower accompanying-plant diversity and simplified accompanying-plant community structure, whereas lightly invaded sites retained relatively higher accompanying-plant diversity. These patterns highlight the importance of maintaining resident vegetation cover and community diversity in lightly invaded areas.

The distribution pattern of *S. canadensis* was associated with both environmental gradients and anthropogenic factors. Environmental analyses highlighted latitude, soil pH, and precipitation of the driest month (Bio14), whereas anthropogenic analyses emphasized floating population density, GDP, transportation route density, freight volume, and motor vehicle ownership as broader anthropogenic gradients associated with human-mediated dispersal and habitat disturbance. MaxEnt modeling identified southern and central Jiangsu as the main climatically suitable areas. This pattern was broadly consistent with the concentration of higher invasion intensity in southern Jiangsu. Field records in northern Jiangsu further suggest that areas with lower predicted climatic suitability should not be excluded from monitoring, as local occurrence and invasion intensity may also be influenced by transportation corridors, habitat disturbance, and propagule pressure.

Overall, the findings support a shift in the management of *S. canadensis* in Jiangsu Province from general control toward region-specific and habitat-specific strategies. Priority should be given to transportation corridors, highly disturbed urban habitats, construction sites, abandoned land, and northern Jiangsu, where field records suggest a need for continued monitoring despite lower predicted climatic suitability. Integrated measures combining early detection, source control, repeated removal before flowering and ecological restoration with locally suitable competitive vegetation may help reduce further spread risk and support the conservation of resident plant-community diversity.

## Figures and Tables

**Table 1:** Regional characteristics of *S. canadensis* invasion in Jiangsu Province.

Characteristics	Group A (*n* = 74)	Group B (*n* = 60)	Group C (*n* = 31)	Overall Average
Plant Height (cm)	109.35 ± 2.82 a	90.62 ± 2.55 b	86.06 ± 4.88 b	98.16
Cover (%)	82.90 ± 1.36 a	64.73 ± 1.63 b	32.66 ± 1.96 c	66.85
Frequency	8.69 ± 0.12 a	7.18 ± 0.23 b	4.40 ± 0.21 c	7.34
Individual density (plants m^−2^)	27.35 ± 1.60 a	11.34 ± 0.62 b	6.08 ± 0.51 c	17.53
Importance Value	0.78 ± 0.02 a	0.48 ± 0.02 b	0.31 ± 0.03 c	0.58
Biomass (g m^−2^)	688.08 ± 27.05 a	350.26 ± 15.36 b	193.51 ± 20.81 c	472.32

Note: Values are means ± SE. Different lowercase letters within the same row indicate significant differences among groups based on one-way ANOVA followed by Tukey’s HSD test at *p* < 0.05.

**Table 2:** Cover of the main accompanying plant species in *S. canadensis*-invaded plots.

Species	Group A (%)	Group B (%)	Group C (%)	Overall Mean (%)
*Setaria viridis* (L.) P. Beauv.	15.61 ± 4.89 b	33.22 ± 2.55 a	27.60 ± 1.64 a	27.99
*Erigeron canadensis* L.	12.69 ± 2.48 b	24.56 ± 2.71 a	14.95 ± 1.93 b	18.69
*Humulus scandens* (Lour.) Merr.	12.94 ± 3.09 b	22.64 ± 2.25 a	22.39 ± 2.26 a	20.59
*Digitaria sanguinalis* (L.) Scop.	9.27 ± 3.12 c	44.12 ± 3.07 a	30.39 ± 2.53 b	31.46
*Symphyotrichum subulatum* (Michx.) G. L. Nesom	18.29 ± 6.18 a	13.34 ± 2.88 a	16.46 ± 2.21 a	15.76
*Setaria faberi* R. A. W. Herrmann	7.89 ± 2.21 b	31.98 ± 3.20 a	32.30 ± 2.85 a	29.27
*Glycine soja* Siebold & Zucc.	5.93 ± 0.85 b	17.07 ± 1.60 a	19.99 ± 1.97 a	16.52
*Causonis japonica* (Thunb.) Raf.	3.83 ± 1.61 b	15.67 ± 3.57 a	10.74 ± 2.14 ab	11.02
*Cynodon dactylon* (L.) Persoon	32.83 ± 6.18 b	41.68 ± 3.01 b	56.60 ± 4.20 a	45.06
*Phragmites australis* (Cav.) Trin. ex Steud.	2.01 ± 1.21 b	16.04 ± 3.30 a	19.21 ± 2.96 a	14.98
*Artemisia lavandulifolia* DC.	2.50 ± 1.47 b	20.50 ± 5.42 a	15.29 ± 1.01 a	14.70

Note: Values are means ± SE. Different lowercase letters within the same row indicate significant differences among groups based on one-way ANOVA followed by Tukey’s HSD test at *p* < 0.05.

**Table 3:** Habitat characteristics of *S. canadensis* occurrence in different habitat groups.

Habitat Group	Percentage of Surveyed Sites (%)	Shannon- Wiener Index	Simpson Index	Importance Value	Accompanying Species Richness
I (*n* = 76)	46.06	0.83 ± 0.05 b	0.49 ± 0.02 b	63.23 ± 2.62 b	3.10 ± 1.28 b
II (*n* = 57)	34.55	0.73 ± 0.05 b	0.44 ± 0.03 b	74.13 ± 2.08 a	2.64 ± 1.01 c
III (*n* = 28)	16.97	0.85 ± 0.09 b	0.50 ± 0.04 ab	62.65 ± 4.48 b	3.34 ± 1.61 ab
IV (*n* = 1)	0.60	1.32	0.71	22.52	4.23
V (*n* = 3)	1.82	1.40 ± 0.05 a	0.75 ± 0.03 a	35.33 ± 18.84 c	4.67 ± 0.58 a

Note: Habitat Group I, dense road-network sites; Habitat Group II, urban disturbed sites; Habitat Group III, park/woodland/orchard sites; Habitat Group IV, agricultural sites; Habitat Group V, riparian or wetland-margin sites. Habitat Group IV contained only one sampled site and was presented descriptively without inclusion in ANOVA or Tukey’s HSD post hoc comparisons. Habitat Group V contained three sampled sites. Values are mean ± SE where applicable. Different lowercase letters within the same column indicate significant differences among statistically compared groups based on one-way ANOVA followed by Tukey’s HSD test at *p* < 0.05.

**Table 4:** Community and diversity indices of accompanying plants across the three cluster-defined *S. canadensis* invasion-intensity groups.

	Group A	Group B	Group C
Total cover of accompanying species	17.83 ± 2.26 c	43.57 ± 3.98 b	63.78 ± 3.28 a
Total number of individuals (accompanying species)	5.24 ± 0.78 c	24.61 ± 3.74 b	44.01 ± 5.45 a
Average height of accompanying species (cm)	24.58 ± 2.29 c	61.87 ± 4.07 a	44.01 ± 1.87 b
Abundance of accompanying species	0.14 ± 0.02 c	0.48 ± 0.04 b	0.69 ± 0.02 a
Total species richness	1.93 ± 0.08 c	2.98 ± 0.13 b	4.36 ± 0.11 a
Simpson index	0.28 ± 0.02 c	0.55 ± 0.02 b	0.68 ± 0.01 a
Shannon–Wiener diversity index	0.43 ± 0.03 c	0.91 ± 0.04 b	1.21 ± 0.03 a
Pielou’s evenness index	0.48 ± 0.03 b	0.86 ± 0.01 a	0.85 ± 0.02 a
Margalef index	0.30 ± 0.02 c	0.58 ± 0.05 b	0.95 ± 0.03 a

Note: Values are means ± SE. Different lowercase letters within the same row indicate significant differences among groups based on one-way ANOVA followed by Tukey’s HSD post hoc test at *p* < 0.05.

**Table 5:** PERMANOVA and related tests of accompanying-species composition among the three *S. canadensis* invasion-intensity groups.

Analysis	Dataset/Comparison	*n*	Statistic	R^2^	*p* Value	Main Result
PERMANOVA	All invaded sites	165	Pseudo-F = 2.97	0.035	<0.001	Significant overall difference in accompanying-species composition among groups, with limited explained variation
Multivariate dispersion test	All invaded sites	165	F = 13.39	—	<0.001	Significant difference in within-group variability among groups
Fisher’s exact test	Sites without accompanying species among groups	165	—	—	0.011	Sites without accompanying species occurred only in Group A
Sensitivity PERMANOVA	Sites with accompanying species only	158	Pseudo-F = 2.64	0.033	<0.001	Significant difference remained after excluding sites without accompanying species

Note: *S. canadensis* was excluded from the accompanying-species matrix because the invasion-intensity groups were defined using its population traits. PERMANOVA was based on Bray–Curtis dissimilarities calculated from accompanying-species cover data. Fisher’s exact test was used to compare the proportion of sites without accompanying species because some expected frequencies were below 5. The sensitivity PERMANOVA was conducted after excluding sites without accompanying species.

**Table 6:** Cross-tabulation of MaxEnt-predicted climatic habitat suitability classes and field-observed invasion-intensity groups of *S. canadensis*.

Region	MaxEnt Suitability Class	Group A	Group B	Group C	Total
Jiangsu Province	Unsuitable	0	18	17	35
Jiangsu Province	Low suitability	12	17	3	32
Jiangsu Province	Moderate suitability	9	5	2	16
Jiangsu Province	High suitability	53	20	9	82
Jiangsu Province	Total	74	60	31	165
Northern Jiangsu	Unsuitable	0	17	17	34
Northern Jiangsu	Low suitability	1	8	3	12
Northern Jiangsu	Moderate suitability	0	1	0	1
Northern Jiangsu	High suitability	0	3	1	4
Northern Jiangsu	Total	1	29	21	51

Note: The MaxEnt suitability classes were defined as unsuitable (<0.185486), low suitability (0.185486–0.340092), moderate suitability (0.340092–0.473936), and high suitability (>0.473936). Group A, Group B, and Group C correspond to the cluster-defined severe-, moderate-, and light-invasion groups, respectively. Values represent the number of field survey sites in each suitability class and invasion-intensity group.

**Table 7:** Description of environmental variables used for MaxEnt model prediction.

Code	Environmental Variables	Units
Bio1	Annual mean temperature	°C
Bio2	Mean diurnal range	°C
Bio3	Isothermality (Bio2/Bio7) (×100)	%
Bio4	Temperature seasonality (standard deviation ×100)	%
Bio5	Maximum temperature of warmest month	°C
Bio6	Minimum temperature of coldest month	°C
Bio7	Temperature annual range (Bio5–Bio6)	°C
Bio8	Mean temperature of wettest quarter	°C
Bio9	Mean temperature of driest quarter	°C
Bio10	Mean temperature of warmest quarter	°C
Bio11	Mean temperature of coldest quarter	°C
Bio12	Annual precipitation	mm
Bio13	Precipitation of wettest month	mm
Bio14	Precipitation of driest month	mm
Bio15	Precipitation seasonality (coefficient of variation)	%
Bio16	Precipitation of wettest quarter	mm
Bio17	Precipitation of driest quarter	mm
Bio18	Precipitation of warmest quarter	mm
Bio19	Precipitation of coldest quarter	mm
Alt	Elevation	m
Slope	Slope	°
Aspect	Aspect	°

## Data Availability

The datasets analyzed in the present study are available from the corresponding author on reasonable request.

## Supplementary Materials

The following supporting information can be downloaded at: https://www.mdpi.com/article/10.3390/plants15142198/s1. Table S1: Occurrence of *S. canadensis* in Jiangsu Province, China; Table S2: Relative contributions of explanatory variables in hierarchical partitioning and their bootstrap-based 95% confidence intervals; Table S3: Variance inflation factor values of explanatory variables retained in the RDA models.
