# Supplementary material for: Patterns, Associated Factors and Plant Diversity Characteristics of Solidago canadensis-Invaded Communities in Jiangsu Province, China
Source: Plants (Basel). 2026 Jul 17;15(14):2198. doi: 10.3390/plants15142198 (PMC13415074; doi:10.3390/plants15142198)
Supplement: Supplementary file 1 [file plants-15-02198-s001.zip › plants-4357285-supplementary.pdf]

Supplementary Table S1. Occurrence of *S. canadensis* in Jiangsu Province, China

City	Total Coverage of Other Species	Total Number of Individuals (Other Species)	Average Height of Other Species	Abundance of Other Species	Total Species Richness	Plant Height	Coverage	Frequency	Number of Individuals	Importance Value	Sample Dry Weight	Simpson's Dominance Index	Shannon-Wiener Diversity Index	Pielou's Evenness Index	Margalef Index	Sample size, n
Xuzhou	65.02±5.09b	36.03±6.33cd	39.44±3.06bcd	0.68±0.05ab	4.47±0.22ab	73.65±6.06e	53.8±4.53cde	5.82±0.42d	8.24±1.03fg	0.33±0.02g	234.95±31.09e	0.72±0.01a	1.29±0.06ab	0.87±0.02a	1.03±0.08a	13
Lianyungang	83.88±5.58a	45.54±3.94bc	44.42±5.13bcd	0.75±0.03a	4.99±0.17a	84.86±6.37de	43.1±6.31e	5.56±0.68d	7.6±1.06fg	0.27±0.02g	220.87±27.44e	0.74±0.01a	1.42±0.05a	0.89±0.02a	1.05±0.04a	10
Suqian	61.57±6.00bc	59.03±15.85ab	47.76±3.5bc	0.76±0.04a	4.25±0.25bc	86.85±5.62de	46.09±5.87de	5.98±0.81d	9.33±1.14fg	0.32±0.02g	180.96±21.6e	0.68±0.02ab	1.24±0.05ab	0.88±0.02a	0.89±0.09a	12
Yancheng	85.25±3.32a	79.88±23.16a	35.03±2.09bcd	0.65±0.06ab	4.4±0.2abc	80.23±4.68de	57.38±6.06bcde	5.65±0.55d	15.48±2.14cdefg	0.38±0.04fg	256.82±26.72de	0.67±0.02ab	0.86±0.03d	0.62±0.03c	0.87±0.05a	8
Huaian	53.5±9.64bcd	19.15±2.38def	50.45±7.54bc	0.55±0.08b	3.95±0.32bc	97.77±11.02bcd	50.15±6.12cde	7.65±1.25abc	14.73±3.62defg	0.45±0.05ef	236.86±48.5e	0.6±0.03bc	1.13±0.08bc	0.85±0.02a	0.88±0.11a	8
Yangzhou	20.82±1.21e	35.8±8.2cd	86.89±4.73a	0.75±0.04a	3.81±0.16c	98.68±4.77bcd	59.83±7.43bcd	6.05±0.62cd	6.43±1.26g	0.37±0.03fg	322.69±46.51de	0.69±0.03ab	1.19±0.04b	0.93±0.01a	0.87±0.13a	10
Taizhou	49.21±6.13cd	23.74±5.29de	50.63±4.27b	0.55±0.05b	3.09±0.25d	111.55±9.84ab	60.64±6.78bc	6.7±0.49bcd	15.35±2.94def	0.5±0.04e	417.1±40.91cd	0.56±0.02c	0.94±0.07cd	0.87±0.02a	0.6±0.08b	14
Zhenjiang	23.49±5.05e	14.97±2.74ef	42.54±5.22bcd	0.32±0.04c	2.88±0.13de	94.96±6.96bcd	83.37±2.98a	8.58±0.28a	28.23±2.53b	0.62±0.02d	531.27±42.57bc	0.5±0.02c	0.84±0.03d	0.82±0.01ab	0.51±0.03bc	12
Nantong	42.23±6.70d	7.66±2.16ef	30.36±4.78de	0.33±0.07c	2.38±0.21ef	91.65±5.47cd	76.42±4.22a	7.71±0.4ab	10.72±1.95efg	0.69±0.05cd	647.62±99.64ab	0.39±0.05d	0.64±0.09e	0.61±0.08c	0.5±0.08bc	16
Nanjing	17.28±5.55e	10.25±4.94ef	17.29±4.15e	0.12±0.04de	1.76±0.16g	125.8±6.39a	81.32±3.73a	8.26±0.3a	39.24±5.68a	0.84±0.03a	543.95±36.44bc	0.23±0.04f	0.34±0.07f	0.39±0.09d	0.21±0.04d	15
Changzhou	18.21±3.67e	4.93±1.26f	30.79±3.71de	0.06±0.01e	2.06±0.16fg	98.21±3.98bcd	82±4.03a	8.55±0.35a	19.76±1.61cd	0.77±0.04abc	647.41±24.72ab	0.32±0.04de	0.5±0.07ef	0.59±0.06c	0.35±0.05cd	15
Wuxi	19±5.90e	3.44±1.01f	31.14±7.54d	0.13±0.04de	1.75±0.13g	106.02±3.84bc	74.8±4.06a	8.24±0.34a	23.24±2.4bc	0.81±0.04ab	687.1±50.1a	0.25±0.03ef	0.37±0.06f	0.41±0.06d	0.27±0.05d	20
Suzhou	24.03±4.39e	6.45±1.61ef	34.23±6.44cd	0.22±0.04cd	1.95±0.16fg	107.46±4.19bc	72.58±3.58ab	8.63±0.31a	18.08±1.28cde	0.74±0.03bc	705.07±38.78a	0.34±0.03d	0.49±0.05ef	0.66±0.07bc	0.31±0.05cd	12
Jiangsu	39.48±2.34	22.97±2.46	39.65±1.87	0.41±0.02	3.00±0.10	98.16±1.97	66.85±1.7	7.34±0.16	17.53±1.03	0.58±0.02	495.4±24.48	0.48±0.02	0.81±0.03	0.69±0.02	0.59±0.03	165

Note: Values are means ± SE. Different lowercase letters indicate significant differences among cities at $P < 0.05$. Sample size, n, indicates the number of quadrat-survey sites in each city.

Supplementary Table S2. Relative contributions of explanatory variables in hierarchical partitioning and their bootstrap-based 95% confidence intervals.

RDA model	Variable	Relative contribution (%)	95% bootstrap CI
Environmental RDA	Latitude	21.71	[16.78, 25.95]
Environmental RDA	Bio03	11.17	[7.94, 15.14]
Environmental RDA	Bio08	4.48	[2.30, 8.05]
Environmental RDA	Bio09	7.88	[4.90, 11.95]
Environmental RDA	Bio10	7.03	[4.62, 10.13]
Environmental RDA	Bio13	6.60	[4.53, 9.86]
Environmental RDA	Bio14	15.57	[11.87, 19.10]
Environmental RDA	Bio18	0.85	[0.25, 3.36]
Environmental RDA	Soil type	6.98	[2.93, 13.94]
Environmental RDA	Soil pH	17.73	[11.60, 24.17]
Anthropogenic RDA	GDP	19.33	[16.72, 22.05]
Anthropogenic RDA	Transportation route density	18.17	[10.58, 26.79]
Anthropogenic RDA	Motor vehicle ownership	12.76	[11.03, 14.82]
Anthropogenic RDA	Freight volume	15.19	[10.38, 19.90]
Anthropogenic RDA	Floating population density	22.25	[15.95, 28.30]
Anthropogenic RDA	Built-up area	12.30	[9.92, 15.04]
Comprehensive RDA	Latitude	23.66	[19.72, 27.03]
Comprehensive RDA	Bio14	17.16	[13.88, 20.38]
Comprehensive RDA	GDP	17.97	[14.08, 21.91]
Comprehensive RDA	Transportation route density	10.10	[5.25, 17.87]
Comprehensive RDA	Motor vehicle ownership	12.63	[9.61, 16.24]
Comprehensive RDA	Floating population density	18.47	[14.06, 23.38]

Note: Relative contributions (%) were estimated using hierarchical partitioning. Values in brackets indicate bootstrap-based 95% confidence intervals estimated by resampling sampling sites.

Supplementary Table S3. Variance inflation factor values of explanatory variables retained in the RDA models.

RDA model	Variable	VIF
Environmental RDA	Latitude	12.98
Environmental RDA	Bio03	5.29
Environmental RDA	Bio08	3.48
Environmental RDA	Bio09	2.35
Environmental RDA	Bio10	5.74
Environmental RDA	Bio13	4.57
Environmental RDA	Bio14	6.95
Environmental RDA	Bio18	2.70
Environmental RDA	Soil type	1.75
Environmental RDA	Soil pH	2.91
Anthropogenic RDA	GDP	83.56
Anthropogenic RDA	Transportation route density	3.67
Anthropogenic RDA	Motor vehicle ownership	80.66
Anthropogenic RDA	Freight volume	16.94
Anthropogenic RDA	Floating population density	5.08
Anthropogenic RDA	Built-up area	89.52
Comprehensive RDA	Latitude	9.31
Comprehensive RDA	Bio14	5.54
Comprehensive RDA	GDP	88.32
Comprehensive RDA	Transportation route density	2.07
Comprehensive RDA	Motor vehicle ownership	64.07
Comprehensive RDA	Floating population density	4.35

Note: VIF values were calculated after standardization of explanatory variables and are reported for the variables retained in each RDA model.
